# Clinical assessment of marginal and bulk fractures and discoloration in posterior composite after 12 to 36 months: A retrospective study

**DOI:** 10.4317/jced.62724

**Published:** 2025-07-01

**Authors:** Vahideh Motamedosanaye, Hadi Akbari, Sara Ziaaddini, Mohammad Mostafazadehbakhtiyary, Atefeh Sadat Langari

**Affiliations:** 1 Restorative Dentistry Department, School of Dentistry, North Khorasan University of Medical Science, Bojnurd, Iran; 2 Addiction and Behavioral Sciences Research Center, North Khorasan University of Medical Science, Bojnurd, Iran; 3 Department of Esthetic and Restorative Dentistry, School of Dentistry, Mashhad University of Medical Sciences, Mashhad, Iran

## Abstract

**Background:**

This retrospective cohort study aimed to evaluate the marginal discoloration, fractures, and bulk fractures of posterior composite restorations over a period of 1 to 3 years.

**Material and Methods:**

A total of 281 restorations, performed by a restorative dental specialist, were assessed using modified United States Public Health Service (USPHS) criteria. Demographic data, occlusal information, and patient habits were collected, along with characteristics of the restoration (composite resin type, adhesive, restoration type, and treated tooth). Data were analyzed using the chi-square test and independent t-test (α = 0.05).

**Results:**

Posterior Gradia, P60, and X-tra Fil were employed in 51.2%, 38.07%, and 14.23% of the restorations, respectively. Most of the restorations (81.5%) received an Alpha score for marginal discoloration, which was significantly associated with composite resin type, colored beverage consumption, oral hygiene, caries risk, and restoration classification (*P*<0.05). Bravo discoloration was more common in patients who received Xtra Fil composite, consumed moderate to high amounts of colored beverages, had moderate to poor oral hygiene, and exhibited high caries risk. Additionally, Bravo discoloration was more frequent in Class II and build-up restorations. Restorations with a Bravo score were found to have significantly higher average restoration age, patient age, and a greater number of missing teeth compared to those with an Alpha score (*P*<0.05). In terms of marginal fractures, 99.6% of restorations received an Alpha score, with no significant associations with the evaluated variables. Furthermore, 99.6% of restorations showed no bulk fractures.

**Conclusions:**

Posterior composite resin restorations demonstrated clinically acceptable performance with respect to marginal discoloration and integrity after 12 to 36 months.

** Key words:**Composite filling, marginal fracture, bulk fracture, discoloration.

## Introduction

Composite resins are frequently utilized in direct, semi-direct, and indirect restorations (1). There is ongoing discussion about the clinical performance of posterior resin composites compared to amalgam. However, evidence supports composites’ long-term use in posterior dentition, showing their capacity to deliver durable and reliable results (1,2).

Long-term clinical studies with follow-up durations of 27 to 30 years have reported only 1% to 2% annual failure rates for posterior resin composite restorations (3-5). Retrospective studies with follow-ups of 3 to 33 years have shown success rates ranging from 48.3% to 87.7% (2,6-10). Treatment failures often necessitate repairs, which require preparing a larger cavity that weakens the tooth structure. Therefore, identifying the risk factors contributing to posterior composite failure is essential to prevent further restorative cycles.

The longevity of these restorations is influenced by various clinical variables, including the restoration’s type, size, and position, as well as the operator’s technique and material selection (11). Demographic factors, such as a patient’s age and gender, and behavioral factors, like caries risk and oral health status, also play a significant role (2,7,12,13). Despite these insights, there are still inconsistencies regarding the specific risk factors for posterior composite failure. Some studies have indicated that factors related to the dentist, patient, and tooth may be more significant than the material selection (9,14). Therefore, further research is warranted to clarify these relationships.

Various clinical parameters are used to evaluate dental restorations. The United States Public Health Service (USPHS) criteria, or the Ryge criteria, is the most widely used scoring system. Several modifications have been proposed to the USPHS criteria to enable more detailed analyses in different types of studies. The modified USPHS criteria assess restorations on a quality scale, with Alpha and Bravo scores indicating clinically acceptable restorations. The distinction between these scores lies in their grading, while Charlie and Delta scores indicate significant changes in the restoration’s condition (15).

This retrospective cohort study evaluates the clinical performance of three types of posterior composite restorations: Gradia Direct (GC, Japan), P60 (3M ESPE, USA), and Xtra fil (VOCO, Germany). The study assesses factors contributing to composite failure, including discoloration and marginal and bulk fractures. The evaluation will be conducted using modified USPHS criteria over a follow-up period of 12 to 36 months.

## Material and Methods

- Sample size calculation

The sample size was determined based on the study by Memarpour Pour *et al*. (16) which reported a Bravo rating of 24% in individuals, with α=0.05 and β=0.2. This calculation resulted in a required sample size of 281 restorations.

The posterior restorations were performed by a restorative specialist in private dental office in Bojnurd, Iran. Class II restorations or build-ups performed on mandibular or maxillary premolars or molars within the last 12 to 36 months were included. Patients were recruited for follow-up visits at the dental offices.

- Data collection

Data collected included patients’ gender, occlusion, type of composite resin (Posterior Gradia Direct, P60, Xtrafil), adhesive (SE bond, Single Bond 2, All Bond 3, Solo Bond M), presence of non-carious cervical lesions (NCCLs), colored beverage consumption, oral hygiene status, caries risk, restoration classification, treated tooth, restoration and patients’ age, and the number of missing teeth (excluding third molars).  Table 1 represent the composition and the respective manufacturer of the composite resins and adhesive resin used in the present study.

A dental explorer and mirror were used for examination by a restorative dentistry specialist. Patients who had unusual incidents, such as abnormal trauma during the follow-up period, or those with parafunctional activity (including bruxism and clenching), were excluded from the study.

The degree of discoloration, marginal fracture, and bulk fracture

The degree of discoloration, marginal fracture, and bulk fracture in the restorations were evaluated based on the modified USPHS criteria (15-17):

Discoloration:

• Alpha: No color change

• Bravo: Slight color change that can be corrected with finishing

• Charlie: Localized color change that cannot be fixed

• Delta: Significant color change in the margin areas that cannot be corrected

Marginal Fracture:

• Alpha: No visible gap, not detectable with a probe

• Bravo: Visible gap detectable with a probe

• Charlie: Gap extending to the dentin

Bulk Fracture:

• Alpha: No bulk fracture

• Delta: Presence of a bulk fracture

- Statistical analysis

Data analysis was conducted using SPSS 23.0 software (IBM Inc., NY, USA). Qualitative variables were analyzed using the chi-square test, while quantitative variables were analyzed using independent t-test. Values less than 0.05 were considered statistically significant.

## Results

In this study, a total of 281 posterior composite restorations were evaluated, with the majority (N=224; 79.7%) performed for women. Regarding the composite resins used, Posterior Gradia, P60, and X-tra Fil were employed in 51.2%, 38.07%, and 14.23% of the restorations, respectively. As for the adhesives, SE Bond, Single Bond 2, All Bond 3, and Solo Bond M were used in 43.06%, 1.4%, 17.79%, and 2.1% of the restorations, respectively. The majority of the restorations were performed on maxillary premolars (53.73%). The average restoration age was 24.6 ± 8.59 months.

 Table 2 presents the distribution of discoloration scores based on restoration and patient factors. Most restorations (81.5%) received an Alpha score, and none were assigned a Delta score. According to the chi-squared test, the discoloration score was not significantly related to factors such as patient gender, adhesive resin type, presence of NCCLs, or the treated tooth. However, it was significantly associated with the type of composite resin (*P*=0.001), colored beverage consumption (*P*<0.001), oral hygiene (*P*<0.001), caries risk (*P*<0.001), and restoration classification (*P*=0.001). Bravo discoloration was more commonly observed in patients who received Xtra Fil composite, had moderate to high consumption of colored beverages, maintained moderate to poor oral hygiene, and exhibited high caries risk. This discoloration score was also more prevalent in Class II and build-up restorations compared to Class I restorations.

Since only one restoration received a Charlie score, it was excluded from the statistical analysis. The t-test results showed that restorations with a Bravo score had significantly higher average restoration age (*P*<0.001), patient age (*P*<0.001), and a higher number of missing teeth (*P*=0.021) compared to those with an Alpha score.

Regarding marginal fractures, as shown in Table 2, 99.6% of restorations received an Alpha score, while 0.4% received a Bravo score. No restorations received a Charlie score. The chi-squared and independent samples t-test revealed no significant relationship between the marginal fracture score and any of the examined variables.

In terms of bulk fractures, 99.6% of posterior composite restorations showed no bulk fracture after 12 to 36 months. Only one restoration (4.0%) exhibited a bulk fracture. This occurred in a woman with a Class I restoration and group function laterotrusive occlusion. The restoration, placed on her mandibular lower premolar, used P60 posterior composite material and SE bond adhesive.

## Discussion

The findings of the present study indicated that, after 12 to 36 months, among the 281 restorations, the majority received an Alpha score for discoloration, with no restorations receiving a Delta score. For marginal fractures, almost all restorations received an Alpha score, and only one restoration received a Bravo score. No restorations were assigned a Charlie score.

Except for the one restoration that underwent a bulk fracture, none of the restorations required repair or replacement, suggesting a high success rate for posterior restorations. This low incidence of fractures could be attributed to the conservative approach in caries removal, the use of a layering technique, and the adequate curing of each layer to minimize polymerization shrinkage (18). Previous studies have demonstrated the good long-term performance of posterior composites, with success rates reported after 27 years (3), 29 years (5), and 30 years (4), although these studies were prospective and controlled. In contrast, the present investigation is a practice-based study focusing on routine dental care. Rodolpho *et al*. (2) also reported an annual failure rate (AFR) below 2.5% for posterior restorations after up to 33 years. Therefore, composite resin appears to be a durable material for posterior restorations.

In the present study, 81.5% of restorations received an Alpha score for marginal discoloration, while only 0.4% received a Charlie score. In contrast, a 3.5-year evaluation by Memarpour *et al*. (16), on the posterior composite Ceram HB Tetric showed that 92% of restorations received a Bravo rating for marginal discoloration, 8% received a Charlie rating, and none received an Alpha rating. This finding contrasts with our study, where most restorations received an Alpha rating for discoloration. The discrepancy may be due to differences in the types of materials used, the study duration, and operator factors. Additionally, the participants in Memarpour *et al*. (16) were children, who may have higher consumption of colorful foods and beverages, which could contribute to increased discoloration.

Marginal discoloration and fractures were not found to be related to the patients’ molar and laterotrusive occlusion. Similarly, Menezes-Silva *et al*. (19) also reported that occlusion was not a significant factor in the failure of Class II restorations when using the Atraumatic Restorative Treatment method with glass ionomer and posterior composite restorations.

The class of cavity restoration also showed a significant correlation with discoloration. Discoloration was more prevalent in Class II and buildup restorations. This could be attributed to the fact that, in these types of restorations, the finishing margins are often located in areas of the tooth that may not have been fully polished, which increases the likelihood of residual adhesive remaining and contributing to discoloration

Regarding marginal fracture, 99.6% of the restorations in the present study received an Alpha score. Memarpour *et al*. (16) reported that 76% of restorations received an Alpha score for marginal adaptation, while 24% received a Bravo score. Similarly, in our study, all restorations scored Alpha or Bravo for marginal adaptation. The marginal and bulk fractures of restorations did not correlate with any of the patients’ or restoration factors, likely due to the low frequency of these failures. However, this lack of correlation may also be influenced by the study duration, properties of the restorative materials, isolation techniques, placement methods, and efforts to minimize polymerization shrinkage.

No relationship was found between the restoration’s marginal discoloration and fractures with the patients’ gender or age. This finding aligns with the study by Burke *et al*. (20). Similarly, Rodolpho *et al*. (2) found that patient gender and age were unrelated with restorative failures. In contrast, other investigations have reported higher failure rates in men (14,21-23). A possible reason for this contradictory findings could be the predominance of women in the present study. Additionally, men are less likely to attend checkup appointments, which could influence the results (2).

Composite resin type was not associated with the marginal fracture score. However, the Bravo score for marginal discoloration was more prevalent in restorations using Xtra Fil composite resin. Both Alpha and Bravo scores are considered clinically acceptable. In a three-year clinical trial by Manhart *et al*. (15), two types of posterior composites, Tetric Ceram and Quixfil, were examined, with both resins yielding comparable results. Similarly, in a split-mouth study, de Almeida Durão *et al*. (24) reported a high and comparable success rate for marginal adaptation using Filtek Bulk Fill (3M ESPE) and Tetric EvoCeram Bulk Fill (Ivoclar Vivadent) in posterior regions after 12 months, according to the USPHS criteria. Therefore, controlled trials suggest that patient and practitioner factors may have a greater influence on the performance of composite resin materials than the choice of resin itself.

The restoration discoloration and fracture were not also related to the adhesive agent used. However, Manhart *et al*. (15) reported marginal discoloration scores of 15% for the composite Quixfil (used with the self-etch adhesive Xeno III) and 13% for Tetric Ceram (used with the total-etch adhesive Syntac Classic). The increased discoloration observed with the self-etch adhesive was attributed to limited marginal sealing of the enamel over time.

In the present study, restoration age was not associated with marginal fractures in composite restorations. This could be attributed to the relatively short duration of our study, and over a longer period, the incidence of fractures might increase. The study by Reda *et al*. (25) found that composites experience minimal marginal changes during the first year. However, restoration age was related to color change. Consistent with this, Manhart *et al*. (15) observed an increase in marginal discoloration over time, with 15% of restorations showing discoloration after three years. This discoloration could be due to the occurrence of marginal microleakage, resulting from the degradation of the adhesive in the marginal area over time. Another contributing factor could be the patient’s diet. In the present cohort, a correlation was found between the consumption of colored beverages and discoloration. This finding aligns with the study by Hassani-Tabatabai *et al*. (26), which demonstrated that the color stability of composites is influenced by the patient’s diet. Additionally, it has been reported that all beverages affect the color stability of resin composites, with coffee and wine having the most significant impact (27).

In the present study, marginal discoloration was significantly associated with the patient’s caries risk, oral hygiene, and the number of missing teeth. However, no such relationship was observed for marginal fracture. Trachtenberg *et al*. (28) also reported that pediatric patients with higher decay, missing, and filled teeth (DMFT) had an increased risk of restoration failure. Similarly, a review study by Demarco *et al*. (12), noted that the risk of restoration failure increases with the number of restored teeth per patient, likely due to the higher caries risk in these individuals. Other studies have identified caries risk, parafunctional habits (9,14,29,30), and the number of remaining teeth in the mouth (31,32) as potential risk factors for restoration failure. Therefore, encouraging patients to attend regular dental check-ups, seek necessary treatments, and follow oral hygiene instructions can significantly enhance the longevity of posterior composite resin restorations.

In the present study, the type of tooth was not found to be related to composite resin discoloration or fracture. This contrasts with the findings of Rodolpho *et al*. (7), who reported that the failure risk for mandibular molars was three times greater than for upper first premolars. In another study, Rodolpho *et al*. (2) found that maxillary molars had a higher risk of failure. The mechanical demands placed on molars are greater than those on premolars. One study indicated that the first molar has the highest susceptibility to the restorative failures (23). Additionally, another investigation showed that after five years, maxillary direct composite restorations are more prone to wear than the mandibular restorations (33). On the other hand, Opdam *et al*. (8) found no difference in the longevity of restorations between molars and premolars for three- or more surface restorations. This discrepancy in various studies may be due to differences in follow-up durations, composite resin materials, and practitioner expertise (general practitioners versus restorative dentistry specialists). In the present study, only 15% of restorations were Class I, which could explain the result. Shorter follow-up durations could also contribute to these findings.

The current study had several limitations, including its relatively short duration and the unequal distribution of restorations across different adhesives, composite resins, and patient genders. Additionally, all restorations were performed in a private practice by a single restorative dentistry specialist, which limits the generalizability of the findings. To better assess the durability of posterior composite resin restorations, further multi-center studies with longer follow-up periods are warranted.

## Conclusions

Within the limitations of the present study, all three composite resins demonstrated acceptable clinical efficacy in terms of marginal discoloration, marginal fracture, and bulk fracture over the follow-up period. However, the Posterior Gradia and P60 composites showed significantly less discoloration, suggesting that the Xtra Fil composite may have a higher susceptibility to staining. The results also indicated that oral hygiene status, caries risk, and consumption of colored beverages were associated with restoration discoloration. Therefore, patients should be advised about the potential for color change in their restorations and the importance of maintaining good oral hygiene to prevent discoloration.

## Figures and Tables

**Table 1:** Composition of materials.

Material	Composition	Manufacturer
P60 Filtek	Resin Matrix: UDMA, Bis-GMA, Bisphenol A Diglycidyl Methacrylate Ethoxylated (Bis-EMA); Filler: 61% inorganic filler (Zirconia/silica)	3M ESPE, USA
Posterior Gradia Direct	Resin Matrix: UDMA, Dimethacrylat, co-monomer; Filler: fluoroaminosilicate glass, pre-polymerized sillica	Gc, Japan
Xtra Fil	Resin Matrix: Bis-GMA, TEGDMA, UDMA, Bis-EMA, Methyl Methacrylate (MMA); Filler: 86% inorganic fillers (not specified)	Voco, Germany
Grandio	Resin Matrix: Bis-GMA, UDMA, TEGDMA; Filler: glass ceramic fillers	Voco, Germany
Clearfil Se Bond	Self-etching primer: 10-Methacryloyoxydecyl Dihydrogen Phosphate (10-MDP), 2-Hydroxyethyl Methacrylate (HEMA), photoinitiator, water; Bonding: 10-MDP, HEMA, Bis-GMA, hydrophilic dimethacrylate, microfiller	Kurary, Japan
Single Bond 2	Etchant: phosphoric acid; Bonding: dimethacrylates, HEMA, polyalkenoid acid copolymer, silica, ethanol, water, photoinitiator	3M ESPE, USA
All Bond 3	Etching: phosphoric acid; Primer: N-Tolylglycin Glycidal Methacrylate (NGT_GMA), free HEMA, Bis_GMA , Biphenyl Dimethacrylate (BPDM), ethanol; Bonding: Bis_GMA, UDMA, TEGDMA, glass filler	Bisco, USA
Solo Bond M	Etching: phosphoric acid; Bonding: Butylated hydroxyltoluene (BHT), HEMA, Bis-GMA, acetone, organic acids	Voco, Germany

**Table 2:** The relationship between the frequency (%) of variables and mean± standard deviation of the restoration’s age, patients’ age and the average missing teeth among the restorations with different discoloration and marginal fracture scores.

Variable		Discoloration score				Marginal fracture score		P value	Total
		Alpha	Bravo	Charlie	P value	Alpha	Bravo		
Gender	Female	186 (81.2)	37 (72.5)	1 (100)	0.346	223 (79.6)	1 (100)	>0.999	224 (79.7)
	Male	43 (18.8)	14 (27.5)	0		57 (20.4)	0		57 (20.2)
Composite resin	Posterior Gradia	116 (50.6)	28 (54.9)	0	0.001*	144 (51.4)	0	0.488	144 (51.2)
	P60	95 (41.5)	11(21.6)	1 (100)		106 (37.9)	1 (100)		107 (38.07)
	X-tra Fil	18 (7.9)	12 (23.5)	0		30 (10.7)	0		40 (14.23)
Adhesive resin	SE bond	180 (78.6)	40 (78.4)	1 (100)	0.754	220 (78.6)	1 (100)	>0.999	121 (43.06)
	Single bond 2	3 (1.3)	1 (2)	0		4 (1.4)	0		4 (1.4)
	All bond 3	40 (17.5)	10 (19.6)	0		50 (17.6)	0		50 (17.79)
	Solo bond M	6 (2.6)	0	0		6 (2.4)	0		6 (2.1)
Non-Carious Cervical Lesion (NCCL)	Absence	222 (96.9)	46 (90.2)	1 (100)	0.104	268 (95.7)	1 (100)	>0.999	269 (95.72)
	Localized	2 (0.9)	1 (2)	0		3 (1.1)	0		3 (1.06)
	Generalized	5 (2.2)	4 (7.8)	0		9 (3.2)	0		9 (3.2)
Colored beverage consumption	High	14 (6.1)	15 (29.4)	0	<0.001*	29 (10.4)	0	>0.999	29 (10.3)
	Moderate	149 (65.1)	29 (56.9)	1 (100)		178 (63.6)	1 (100)		179 (63.7)
	Low	66 (28.8)	7 (13.7)	0		73 (26)	0		73 (25.9)
Oral Hygiene	Good	116 (50.7)	11 (21.6)	1 (100)	<0.001*	127 (45.5)	1 (100)	0.477	128 (45.55)
	Moderate	109 (47.6)	38 (74.5)	0		147 (52.5)	0		147 (52.31)
	Poor	4 (1.7)	2 (3.9)	0		6 (2.1)	0		6 (2.1)
Caries Risk	Low	134 (58.5)	16 (31.4)	0	<0.001*	150 (53.6)	0	0.466	150 (53.3)
	High	95 (41.5)	35 (68.6)	1 (100)		130 (46.4)	1 (100)		131 (46.6)
Restoration Classification	Class I	38 (16.6)	5 (9.8)	0	0.001*	43 (15.4)	0	0.299	43 (15.3)
	Class II (Mesial)	81(35.4)	13 (25.5)	0		94 (33.6)	0		94 (33.4)
	Class II (Distal)	53 (23.1)	7 (13.7)	0		60 (21.4)	0		60 (21.35)
	Class II (Mesial, Distal)	30 (13.1)	6 (11.8)	1 (100)		36 (12.8)	1 (100)		37 (13.16)
	Vital Build-up	14 (6.1)	8 (15.7)	0		22 (7.9)	0		22 (7.8)
	Non-Vital Build-up	13 (5.7)	12 (23.5)	0		25 (8.9)	0		25 (8.9)
Type Of Tooth	Maxillary Premolar	126 (55.02)	25 (49)	0	0.58	151 (53.9)	0	0.463	151 (53.73)
	Mandibular Premolar	29 (12.66)	13 (25.5)	0		42 (15)	0		42 (14.94)
	Maxillary Molar	31 (13.53)	8 (15.7)	0		39 (14)	0		39 (13.87)
	Mandibular Molar	43 (18.77)	5 (9.8)	1 (100)		48 (17.1)	1 (100)		49 (17.43)
Average restoration age		23.46±8.45	29.96±6.78	15±0.0	<0.001**	24.67±8.55	15±0.0	0.254	24.6±8.59
Average patients' age		25.25±9.86	31.7±12.89	34±0.0	<0.001**	26.43±10.75	34±0.0	0.482	26.4±10.77
Average number of missing teeth		0.70±1.54	1.08±1.59	1±0.0	0.021**	0.77±1.55	0.0±0.0	0.594	0.77±1.55
Total		229 (81.5)	51 (18.1)	1 (0.4)		280 (99.6)	1 (0.4)		

* Values less than 0.05 represent a significant difference between the frequency of variables’ classification and the discoloration scores according to the chi-square test. ** Values less than 0.05 represent a significant difference between the restoration’s age, patients’ age, and the average number of missing teeth among the restorations with different discoloration scores, according to the independent samples t-test.

## Data Availability

The datasets used and/or analyzed during the current study available from the corresponding author on reasonable request.
